# Calcium Oxide Matrices and Carbon Dioxide Sensors

**DOI:** 10.3390/s120505896

**Published:** 2012-05-08

**Authors:** Tercio Bezerra Correia Terencio, Valter Bavastrello, Claudio Nicolini

**Affiliations:** 1 Laboratories of Biophysics and Nanobiotechnology, Department of Medical Science, University of Genova, Via Pastore 3, Genova 16132, Italy; E-Mails: posne@hotmail.com (T.B.C.T.); bavastrello_valter@yahoo.it (V.B.); 2 Nanoworld Institute, Fondazione EL.B.A. Nicolini, Largo Redaelli 7, Pradalunga, Bergamo 24100, Italy

**Keywords:** sensors, carbon dioxide, calcium oxide, composites, nanogravimetry

## Abstract

Homogeneous matrices of calcium oxide (CaO) were prepared by mixing this material with polyethylene glycol (PEG) acting as malleable inert support in order to obtain processable composites. Preliminary tests were carried out to assess the best concentration of CaO in the composite, individuated in the CaO/PEG weight ratio of 1/4. Experimental data highlighted that the composite was able to selectively detect carbon dioxide (CO_2_) via a nanogravimetric method by performing the experiments inside an atmosphere-controlled chamber filled with CO_2_. Furthermore, the composite material showed a linear absorption of CO_2_ as a function of the gas concentration inside the atmosphere-controlled chamber, thus paving the way for the possible use of these matrices for applications in the field of sensor devices for long-term evaluation of accumulated environmental CO_2_.

## 1. Introduction

The relentless World population increase is resulting in significant expansions of urban development and agricultural, economic and industrial activities. Furthermore, the World population increase is also responsible for accelerating deforestation and habitat destruction to accommodate the need for open space. Accompanying this are increased emissions of gases from combustion of fossil fuels, industrial processes, and agriculture resulting in consequent changes in the chemical composition of the atmosphere.

One of the most important drawbacks concerning the increased emissions of gases such as carbon dioxide (CO_2_) into the atmosphere is the possible or potential effects of accumulation of these gases at a faster rate than natural environmental removal processes, also taking into account that some 20–40% of anthropogenic fossil fuel CO_2_ emissions can remain in the atmosphere for periods of up to tens of thousands of years [1–3].

The extra accumulation of CO_2_ above levels that can be tolerated and balanced by the self-regulating processes and dynamics of the atmosphere may have direct biological effects, or taken as a whole, they may also influence the Earth's climate [4] by causing changes in the atmospheric re-radiative effect (the “greenhouse effect”) resulting in atmospheric warming, global temperature increases, and changes in wind events and precipitation patterns.

Recently regional and local studies related to CO_2_ accumulation show that values of twice the background value, estimated in about 370 ppmv, are usually found in urban centres of the main cities, where a “dome effect” has been highlighted [5–9]. Many works show that CO_2_ concentration values around and over 1,000 ppmv in indoor sites are often assessed, depending on both internal sources and external air pollution, coupled with low air change levels [10–15].

The common denominator of the above mentioned investigations is that point by point measured differences range between tens and hundreds of ppmv, and differential values are more relevant than the absolute determined value which, on the contrary, is the basic parameter when background values of remote sites are required.

The instruments generally employed in these determinations basically consist of infrared detectors performing a continuous plotting in the site under investigation. The data obtained with such devices are characterised by a good degree of accuracy and precision but they cannot be used for extensive mapping work because of the large number of expensive devices and specialised people which would have to be involved. In fact, most field works cover only a few weeks of investigation.

Diffusive sampling techniques, also described as passive sampling techniques, are the cheapest and easiest way to perform extensive monitoring campaigns both in spatial and temporal terms [16]. In fact, diffusive samplers do not need any energy source, are small and silent, quite inexpensive and people in charge of using these methods of sampling require only a little training for the positioning and removal of the devices. Moreover, if a long-term sampling strategy is adopted [17–19], costs can be further lowered. In fact, it allows evaluating the time-weighted average concentration of pollutants over statistically significant periods, in the order of several days or weeks. The goal of the long-term strategy is that the data are unaffected from short time episodes due to the diurnal variability of mobile sources, to episodic meteorological phenomena, to accidents or, for example, in the case of industrial plants and big air-conditioning apparatus, to the variability of the production cycle and the external air mixing rate. Furthermore, passive devices are useful to perform sampling campaigns in remote sites, where energy sources are not disposable.

Taking into account all the considerations discussed so far, in this work we thus propose an alternative long-term sampling method for the determination of environmental CO_2_ accumulation by taking advantages of the properties of CaO to be carbonated by this gas. For this purpose, we studied the variation of mass connected to the carbonation process by using an atmosphere-controlled chamber, in order to assess the quantity of gas absorbed in relation to the concentration of environmental CO_2_ by a fixed amount of composite.

## 2. Materials and Methods

All reagents for the preparation of the composite were purchased from Sigma Aldrich. Specifically, CaO anhydrous powder used for the matrices had a purity of 99.99%, while PEG in chips had an average molecular weight ranging between 1,900 and 2,000.

### 2.1. Calcium Oxide Matrices

We studied the best processability by trying several CaO/PEG weight ratios since the micrometric-sized granulometry of the CaO fully hindered the adhesion of this material onto the substrates. We therefore tested different concentrations of CaO and PEG and the experimental results highlighted a CaO/PEG weight ratio of 1/4 as the best processable composite. For the preparation of the matrices we ground the CaO powder along with the PEG in chips by means of a ball mill in order to obtain a highly homogeneous mixture. Since the composite melted at low temperature (50 °C), it was possible to prepare the various substrates by simply depositing the right quantity of material and subsequently performing a soft heating process for a few seconds, in order to make the composite fully adhere to the substrates.

### 2.2. Sensing Apparatus

**Figure 1.**
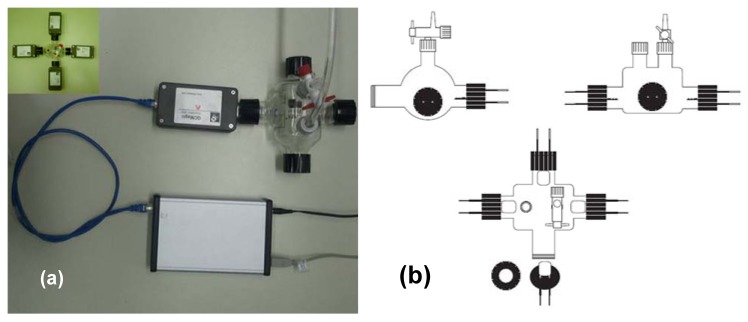
Experimental set up used for the determination of CO_2_: (**a**) Single quartz configuration and multi quartz configuration (inset of Figure 1(a)); (**b**) Schematic of the atmosphere-controlled chamber.

We tested the sensing properties of the composite materials via a nanogravimetric method by using a homemade glass chamber of 340 mL in volume. The homemade chamber, illustrated in Figure 1, was provided with four input sockets able to arrange up to four quartzes at the same time, besides inlet and outlet valves to feed and empty the gas.

**Figure 2.**
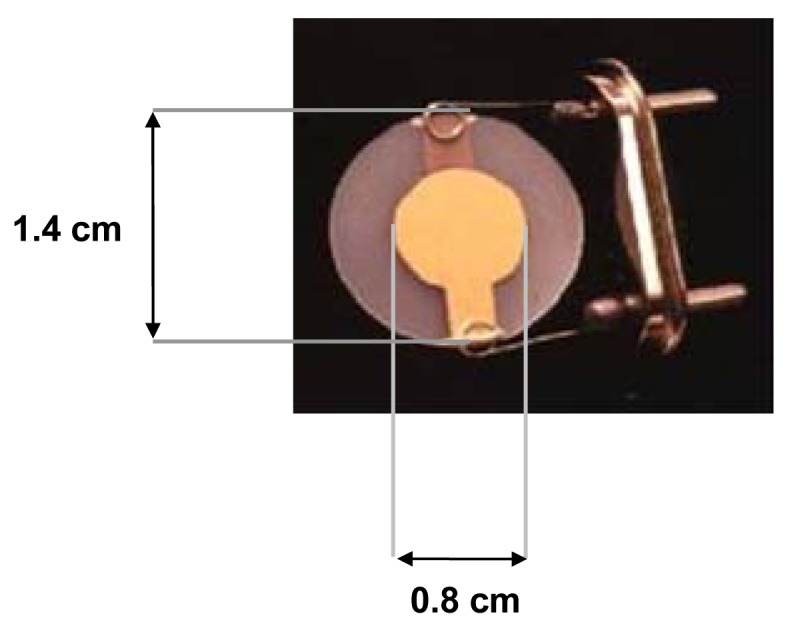
Illustration of the quartz used for the nanogravimetric acquisitions.

For the experiments we used quartzes from International Crystal Manufacturing Co. (Oklahoma City, OK, USA). The quartzes had a diameter of 1.4 cm provided with chromium-gold (chromium 10 nm in thickness and gold 100 nm in thickness) electrodes of 0.855 cm in diameter as shown in Figure 2, and operated at a fundamental frequency of 9.5 MHz. Quartzes were directly connected to a nanogravimeter QCMagic R3 (Elbatech, Ashdod, Isreal) supplied by computer and provided with a USB card for data acquisition.

The apparatus containing the nanogravimetric sensing device was employed in different tests to firstly verify the selectivity and then the sensitivity towards CO_2_ of the composite deposited on quartz electrodes, and all experiments were performed by depositing 1.5 mg of accurately weighed composite.

**Figure 3.**
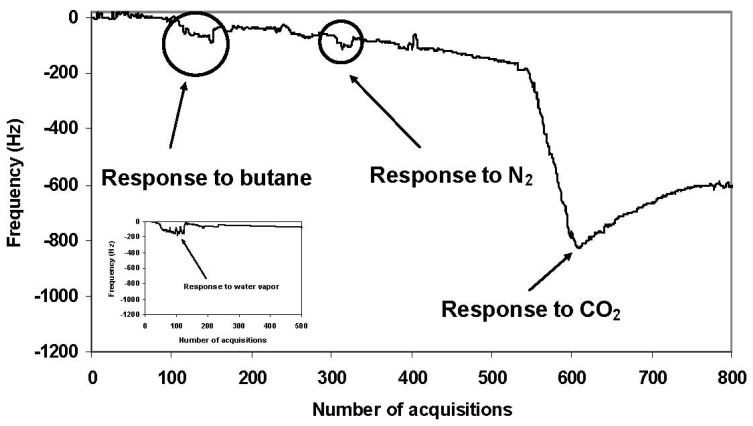
Nanogravimetric acquisitions obtained by feeding the atmosphere-controlled chamber with butane, N_2_, and CO_2_, respectively. Experimental data showed the sensor device was able to detect selectively CO_2_, while other gases were not detected.

Preliminary tests were carried out to study the selectivity of the sensor towards water vapor in view of the possible interferences due to environmental humidity. The experimental results highlighted the fact that water vapor did not affect the signal, probably because the high PEG concentration in the composite was able to reduce the water absorption by CaO (see inset of Figure 3). We consequently tested the response of the sensor device to study the selectivity *versus* different gases such as CO_2_, nitrogen (N_2_) and butane, while the related sensitivity was obtained by filling the atmosphere-controlled chamber with different quantities of CO_2_. All gases were fed into the chamber at a flow rate of 5 L/h, controlled by means of a flowmeter placed between the outlet of the gas cylinder and the chamber itself.

## 3. Results and Discussion

### 3.1. Test of Selectivity

We performed the selectivity tests by introducing two quartzes acting as reference and sample inside the atmosphere-controlled chamber, respectively. After an initial period of adjustment in order to obtain a constant baseline we fed, besides CO_2_, different gases into the chamber at a flow of 5 L/h for 20 minutes. It is important to highlight the standardized feeding system was set up to avoid any inconvenience due to possible variations of pressure inside the atmosphere-controlled chamber in order to eliminate any different detectable signal not connected to absorption of gas into the matrix.

The experimental results highlighted the sensor device was able to detect only CO_2_ while other gases were not detected, as shown in Figure 3, where a clear peak was present only in the first case. The selectivity was assured by the carbonation of CaO shown in Equation (1), where additions of CO_2_ to the composite mainly produced calcium carbonate (CaCO_3_):

(1) $$\text{CaO}+{\text{CO}}_{2}⇄{\text{CaCO}}_{3}$$

The carbonation of CaO was thus responsible of the more evident variation in mass recorded by nanogravimetry with respect to simple absorption phenomena occurring for other gases, demonstrating the contribution due to mere absorption processes into the matrix of the composite was insufficient to generate detectable signals.

### 3.2. Test of Sensitivity

**Figure 4.**
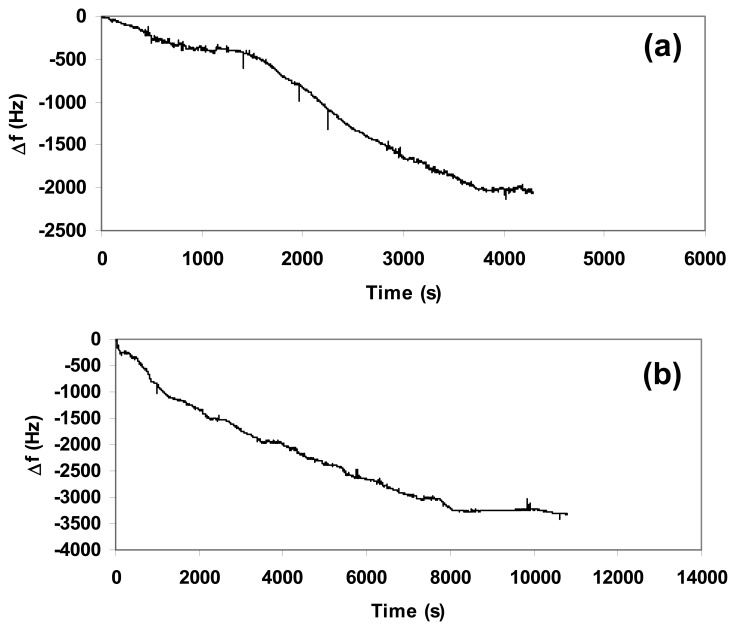

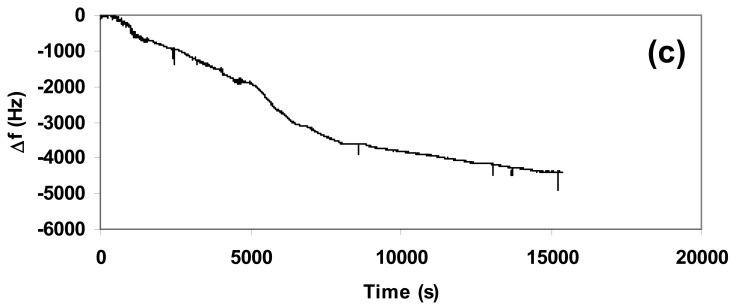
Variation of frequency due to carbonation of CaO obtained from different quantities of CO_2_ blown into the atmosphere-controlled chamber: (**a**) 250 mg; (**b**) 450 mg; (**c**) 650 mg.

We carried out sensitivity tests to verify the response of the composite towards different concentrations of CO_2_. In order to have the best reproducibility we deposited the same quantity (1.5 mg) of composite over the quartz used in each experiment and set the flow at 5 L/h, corresponding to 2.5 mg/s of CO_2_. Figure 4(a–c) showed the nanogravimetric spectra related to the presence of 250 mg, 450 mg, and 650 mg of CO_2_ inside the atmosphere-controlled chamber.

The study of spectra highlighted a proportional absorption of CO_2_ into the composite matrix in function of the gas concentration at a fixed quantity of deposited material, since the variation in frequency due to the absorption from the beginning up to the stabilization of the quartz showed a linear increment. The related variations in frequency (Δf) are summarized in Table 1 for a clear vision.

**Figure 5.**
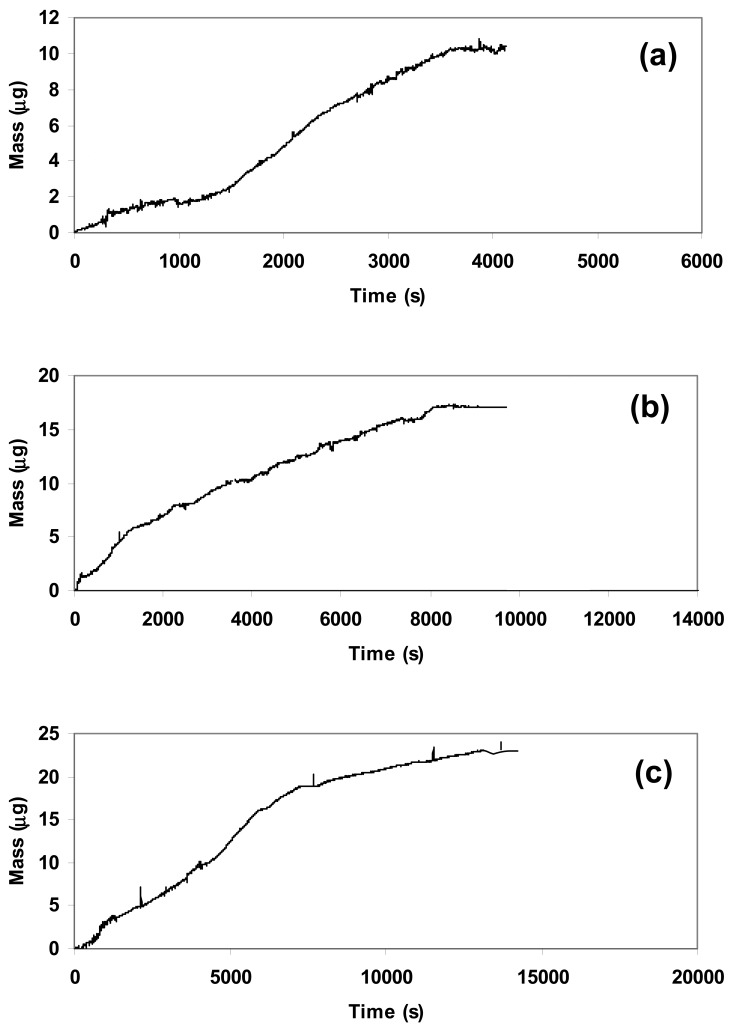
Variation of mass due to carbonation of CaO obtained from different quantities of CO_2_ fed into the atmosphere-controlled chamber: (**a**) 250 mg; (**b**) 450 mg; (**c**) 650 mg.

The increment in quantity of gas inside the atmosphere-controlled chamber was also responsible for the longer time necessary to carry out the absorption process, since the reaction between CaO and CO_2_ was not instantaneous. According to Equation (2):

(2) $$\Delta \text{m}=-\frac{\Delta \text{f}\times 10^{6}}{2.26\times {{\text{f}}_{0}}^{2}}$$

where **Δm** is the variation of mass, **f_0_** is the initial frequency, **Δf** the variation of frequency, we calculated the increment in mass related to the gas absorption onto the composite matrix, as illustrated in Figure 5(a–c).

**Figure 6.**
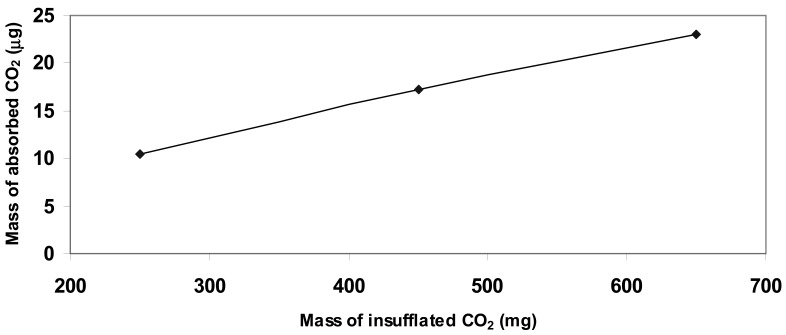
Linear absorption of gas into the composite matrix in relation to the environmental concentration of CO_2_.

The experimental data highlighted a linear behavior referred to the quantity of CO_2_ inside the chamber and the increment in weight of the deposited matrix, as summarized in Table 2 and plotted in Figure 6.

The study of the recorded spectra also showed the carbonation of CaO proceeding on the composite was not fast enough to perform short-time determinations of environmental CO_2_, but the constant formation of CaCO_3_ may find interesting applications for the determination of atmospheric accumulations of this gas in long-time sampling, *i.e.*, providing important information related to CO_2_ concentration values in indoor sites in relation to internal sources and external air pollution, in addition to low air change levels to assess the quality of air in areas used for human activities.

The experimental results thus highlighted CaO may be successfully used for the fabrication of innovative matrices capable of performing long-term sampling of environmental CO_2_ when mixed with a malleable support such as PEG to form a homogenous composite, since the specific reaction of carbonation occurring on the composite materials provide good selectivity and revealed linear proportionality towards the concentration of environmental CO_2_.

## 4. Conclusions

We prepared homogeneous matrices of CaO by mixing this material with a malleable inert support in order to obtain low-temperature processable composites. We experimentally tried different concentrations of CaO inside the composite in order to obtain the best malleability, settling on a CaO/inert support weight ratio of 1/4.

Experimental data showed the composite material was able to selectively detect CO_2_ by a nanogravimetric method, since the carbonation reaction occurring on the composite materials was able to generate perceptible variations in mass, while mere phenomena of absorption associated with other gases were not sufficient to produce detectable nanogravimetric signals. Experimental data also highlighted that water vapor did not affect the signal, probably due to the fact the high concentration of PEG in the composite affected the water absorption by CaO. Furthermore, the composite showed a linear absorption of CO_2_ in function of different concentrations of this gas inside the atmosphere-controlled chamber.

Even though the reaction of carbonation occurring on the composite was not fast enough to perform immediate determination of gas concentrations, CaO mixed with a malleable support such as PEG to form a homogenous composite may be successfully employed for the fabrication of innovative matrices capable to perform long-term sampling of environmental CO_2_, specially where indoor human activities associated to both environmental pollution and scarce air refreshment may produce health problems.

## Figures and Tables

**Table 1.** Variation of frequency in relation to the quantity of CO_2_ present in the atmosphere-controlled chamber obtained by depositing the same quantity of composite.

**Amount of composite (mg)**	**Amount of CO_2_ (mg)**	**Variation of frequency Δf (Hz)**
1.5 ± 0.1	250 ± 0.5	2,000 ± 12
1.5 ± 0.1	450 ± 0.5	3,200 ± 12
1.5 ± 0.1	650 ± 0.5	4,300 ± 12

**Table 2.** Variation of mass in relation to the quantity of CO_2_ present in the atmosphere-controlled chamber obtained by depositing the same quantity of composite.

**Mass of composite (mg)**	**Mass of CO_2_ (mg)**	**Variation of mass Δm (μg)**
1.5 ± 0.1	250 ± 0.5	10.4 ± 0.007
1.5 ± 0.1	450 ± 0.5	17.2 ± 0.007
1.5 ± 0.1	650 ± 0.5	23.0 ± 0.007
